# Exploring the Clinical Utility of Gustatory Dysfunction (GD) as a Triage Symptom Prior to Reverse Transcription Polymerase Chain Reaction (RT-PCR) in the Diagnosis of COVID-19: A Meta-Analysis and Systematic Review

**DOI:** 10.3390/life11121315

**Published:** 2021-11-29

**Authors:** Khang Wen Pang, Sher-Lyn Tham, Li Shia Ng

**Affiliations:** Department of Otolaryngology-Head and Neck Surgery, National University Hospital, Singapore 119228, Singapore; sherr1996@hotmail.com (S.-L.T.); li_shia_ng@nuhs.edu.sg (L.S.N.)

**Keywords:** COVID-19, SARS-CoV-2, ageusia, taste, gustatory dysfunction

## Abstract

Background: The diagnosis of COVID-19 is made using reverse transcription polymerase chain reaction (RT-PCR) but its sensitivity varies from 20 to 100%. The presence of gustatory dysfunction (GD) in a patient with upper respiratory tract symptoms might increase the clinical suspicion of COVID-19. Aims: To perform a systematic review and meta-analysis to determine the pooled sensitivity, specificity, positive likelihood ratio (LR+), negative likelihood ratio (LR−) and diagnostic odds ratio (DOR) of using GD as a triage symptom prior to RT-PCR. Methods: PubMed and Embase were searched up to 20 June 2021. Studies published in English were included if they compared the frequency of GD in COVID-19 adult patients (proven by RT-PCR) to COVID-19 negative controls in case control or cross-sectional studies. The Newcastle-Ottawa scale was used to assess the methodological quality of the included studies. Results: 21,272 COVID-19 patients and 52,298 COVID-19 negative patients were included across 44 studies from 21 countries. All studies were of moderate to high risk of bias. Patients with GD were more likely to test positive for COVID-19: DOR 6.39 (4.86–8.40), LR+ 3.84 (3.04–4.84), LR− 0.67 (0.64–0.70), pooled sensitivity 0.37 (0.29–0.47) and pooled specificity 0.92 (0.89–0.94). While history/questionnaire-based assessments were predictive of RT-PCR positivity (DOR 6.62 (4.95–8.85)), gustatory testing was not (DOR 3.53 (0.98–12.7)). There was significant heterogeneity among the 44 studies (I^2^ = 92%, *p* < 0.01). Conclusions: GD is useful as a symptom to determine if a patient should undergo further testing, especially in resource-poor regions where COVID-19 testing is scarce. Patients with GD may be advised to quarantine while repeated testing is performed if the initial RT-PCR is negative. Funding: None.

## 1. Introduction

COVID-19 is now recognised as an infection with protean multi-system manifestations, including severe pneumonia [1], myocardial dysfunction [2,3], diarrhoea [4,5], thromboembolism [6,7,8], acute cerebrovascular disease [6,9], encephalitis [6,10], Guillain–Barré syndrome [6,11], olfactory dysfunction (OD) and gustatory dysfunction (GD) [6,12,13,14,15], and a Kawasaki-like syndrome in children [16,17].

A multipronged surveillance and containment strategy consisting of active detection of COVID-19 cases, contact tracing and early isolation [18,19,20,21], coupled with social distancing [22,23,24], appear to be effective in controlling the COVID-19 outbreak. However, the major constraints [25,26,27,28] to blanket testing of populations are trained personnel to administer the swabs and run the tests, cost, materials (swab sticks, sample media, reagents) and turnaround time for the reverse transcription polymerase chain reaction (RT-PCR) test on respiratory samples. In addition, the sensitivity of the “gold standard” RT-PCR ranges from 20 to 100% depending on the time from exposure and symptom onset [29], and clinicians should not rely on a single negative RT-PCR test to exclude COVID-19 if clinical suspicion is high [29,30].

While COVID-19 infections present most commonly as an acute upper respiratory tract infection (URTI) (fever, cough, sore throat, myalgia) [31], there are a number of peculiar symptoms which differentiate it from other viruses. It has been shown that OD and GD are common among COVID-19 patients [32,33,34]. Carrillo-Larco et al. [35] found that the prevalence of GD among COVID-19 patients in 6 included studies varied widely from 5 to 89%, with heterogeneous definitions of GD. Smell refers to the perception of odour by the olfactory fibres in the roof of the nasal cavity [36] while taste refers to the perception of salty, sweet, sour, bitter and umami by the tongue carried by cranial nerves VII, IX and X [37]. On the other hand, flavour is a complex perception and refers to the combination of smell, taste and trigeminal sensation (pain, tactile and temperature) [36]. While there is abundant research on OD including the use of smell tests (such as Sniffin’ Sticks [38], University of Pennsylvania Smell Identification Test [UPSIT] [39] and Connecticut Chemosensory Clinical Research Center orthonasal olfaction test (CCCRC) [40,41]) in COVID-19 patients, GD is less well studied.

Taste is important for quality of life, appetite, satiety and is part of a defence mechanism against hazards [42]. More importantly, if GD as a symptom possesses high diagnostic value, it may be used in isolation or in combination with other specific symptoms as part of a screening questionnaire to determine if a patient should undergo further testing, especially in resource-poor regions where COVID-19 testing is scarce. It may also be used to determine the level of clinical suspicion of COVID-19, so that appropriate isolation measures are instituted before repeated testing is performed if the first RT-PCR is negative [30]. Post-viral OD is well established among viral upper respiratory tract infections [43,44,45,46], which reduces its diagnostic value in differentiating COVID-19 from other viruses. Therefore, this study aims to determine if GD, with or without OD, may be used as a discriminatory criterion instead to predict a patient’s COVID-19 status.

Published meta-analyses of the diagnostic value of GD in COVID-19 are sub-optimal. Hoang et al. [47] only pooled data for one subgroup analysis in April 2020, reporting an odds ratio (OR) of 12.7 of GD in COVID-19 versus patients with acute respiratory infections without detectable virus, including only 2 studies with a total of 392 patients. Liou et al. [48] performed a meta-analysis in May 2020 and reported the sensitivity, specificity, positive predictive value, negative predictive value and accuracy of combined taste or smell alteration in the prediction of COVID-19 across 6 studies but did not report statistics for GD (with or without OD).

The study aims to perform a systematic review and meta-analysis to determine the pooled sensitivity, specificity, positive likelihood ratio (positive LR), negative likelihood ratio (negative LR) and diagnostic odds ratios of using gustatory dysfunction as a triage symptom prior to RT-PCR in the diagnosis of COVID-19.

## 2. Materials and Methods

### 2.1. Definition of GD

For the purposes of this meta-analysis, GD is defined as the presence of quantitative (ageusia (complete loss of taste), hypogeusia (diminished sense of taste) and hypergeusia (increased gustatory sensitivity)) or qualitative dysfunction (dysgeusia (distorted taste perception) and phantogeusia (phantom taste perception)) or a combination of the above [36,42], either reported, measured, or both. The list of abbreviations can be found in Table A3.

### 2.2. Systematic Review Protocol

The methodology follows a similar study previously published by the author on the clinical utility of OD in COVID-19 [49]. The review protocol was not registered on any registry.

The Preferred Reporting Items for Systematic reviews and Meta-analyses (PRISMA) Statement [50] was used to structure the systematic review and meta-analysis as shown in Table A4. No ethics approval was required.

### 2.3. Information Sources and Search Strategy

Studies were eligible if they were indexed on PubMed or Embase. Cochrane Central Register of Controlled Trials (CENTRAL) was not searched as trials were irrelevant to the present study. The search was performed on 20 June 2021. The search strategy is included in Table A1 and was not limited by publication date as some articles are indexed prior to publication.

### 2.4. Study Selection and Data Collection

Screening of titles and abstracts was performed by 2 independent researchers (K.W.P., S.L.T.) to determine if the studies met the inclusion criteria. If abstracts were not available, the full text was retrieved and analysed. Any disagreements between the 2 researchers were resolved by discussion and by consulting a third, senior researcher (L.S.N.), to determine if the studies met the inclusion criteria. Duplicate studies were removed by Endnote X9 and then by hand. Data was extracted from eligible studies into Excel sheets by 1 researcher (K.W.P.) and then cross-checked by a 2nd researcher (S.L.T.). These included the author, year of publication, study design, country, GD testing method, COVID-19 testing method and number of cases reporting GD among COVID-19 positive and negative patients. All clarifications with authors were made via email.

The Newcastle-Ottawa scale [51] was used to assess the methodological quality of the included studies. Each item was allocated 1 point except for the item on the “Comparability of cases and controls on the basis of age and URTI symptoms”, which was allocated 2 points. The studies were classified as having low (7–9 points), moderate (4–6 points) and high risk of bias (1–3 points). Assessment was performed by 2 independent researchers (K.W.P., S.L.T.) and any disagreements were resolved by consulting the senior researcher (L.S.N.).

### 2.5. Inclusion and Exclusion Criteria

We compared the frequency of GD in adult patients (at least 18 years) stratified by COVID-19 test results using the reverse transcription polymerase chain reaction (RT-PCR). Studies were included if they compared the frequency of GD in COVID-19 positive patients (proven by RT-PCR) to COVID-19 negative controls in case control or cross-sectional studies. Appropriate controls were defined as patients suspected of having COVID-19 infection or fulfilled local guidelines for COVID-19 testing but were COVID-19 negative on RT-PCR testing. Only studies published in English were included.

### 2.6. Statistical Analysis

R Studio version 1.4.1717 [52] and R version 4.1.0 [53] were used for all statistical analyses. The packages meta [54], mada [55] and dmetar [56] were used in the analyses. Principal summary measures were pooled sensitivity, specificity, positive likelihood ratio (LR), negative LR and diagnostic odd ratios (DOR). All data were presented as effect estimates with 95% confidence intervals in parenthesis, and accompanying forest plots when appropriate. Heterogeneity among studies was tested using the Cochran’s Q test and I^2^. A random-effects model was used if I^2^ > 50%. Forest plots were generated to summarise the results. Funnel plots and Peters’ tests were used to detect any publication bias.

### 2.7. Subgroup Analyses

Subgroup analyses was performed using a random-effects model as follows.

#### 2.7.1. Comparison 1

Group A: studies with either high risk of bias on the Newcastle-Ottawa scale, in which GD symptoms were not explicitly asked for or tested, or in combination.

Group B: studies with low to moderate risk of bias on the Newcastle-Ottawa scale and in which GD symptoms were explicitly asked for or tested.

#### 2.7.2. Comparison 2

Group C: studies utilising questionnaire-based assessments of GD.

Group D: studies utilising gustatory testing.

## 3. Results

Using the search strategy, 3244 references were retrieved, with 1187 studies from PubMed and 2057 studies from Embase. Following which, 816 duplicates were automatically removed by Endnote while 179 duplicates were identified and removed by hand.

**Figure 1:**
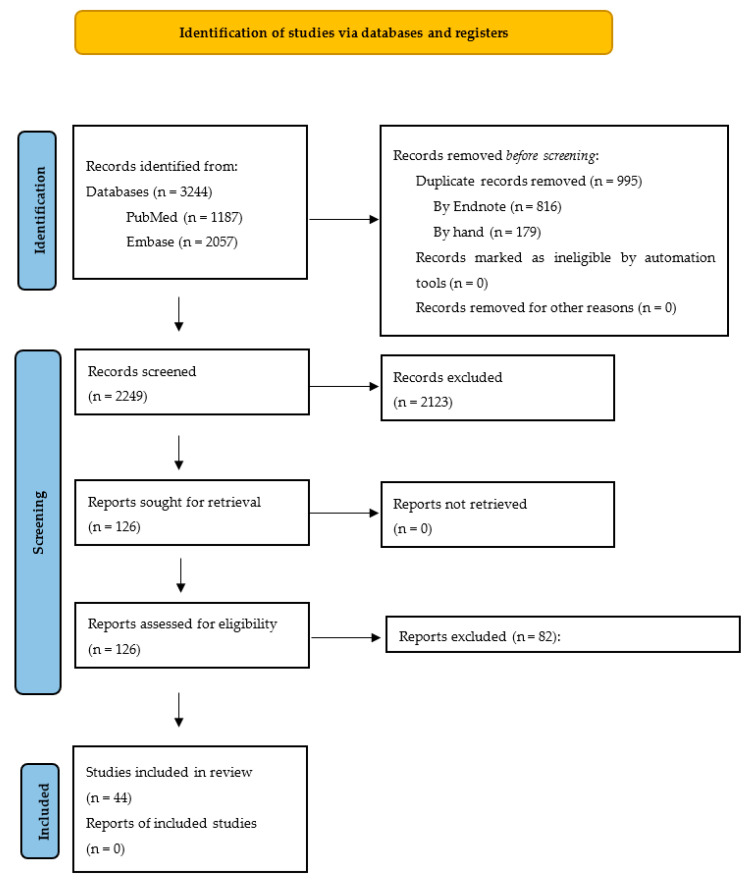
Flow diagram [50]. Reports excluded (n = 82): 7 papers used COVID-19 serology instead of RT-PCR [57,58,59,60,61,62,63]; 5 papers did not include sufficient raw data, despite contacting authors via email [64,65,66,67,68]; 35 papers did not provide a comparison group (COVID-19 negative patients) [69,70,71,72,73,74,75,76,77,78,79,80,81,82,83,84,85,86,87,88,89,90,91,92,93,94,95,96,97,98,99,100,101,102,103]; 4 papers used inappropriate comparison groups for this meta-analysis [104,105,106,107]; 30 papers grouped olfactory and gustatory dysfunction together [108,109,110,111,112,113,114,115,116,117,118,119,120,121,122,123,124,125,126,127,128,129,130,131,132,133,134,135,136,137]; 1 paper included paediatric cases [138].

Furthermore, 2123 articles were excluded based on their titles and abstracts and 82 of the remaining 126 articles were excluded for reasons as described in Figure 1. The remaining 44 articles were included in the meta-analysis.

### 3.1. Study Characteristics

**Figure 2:**
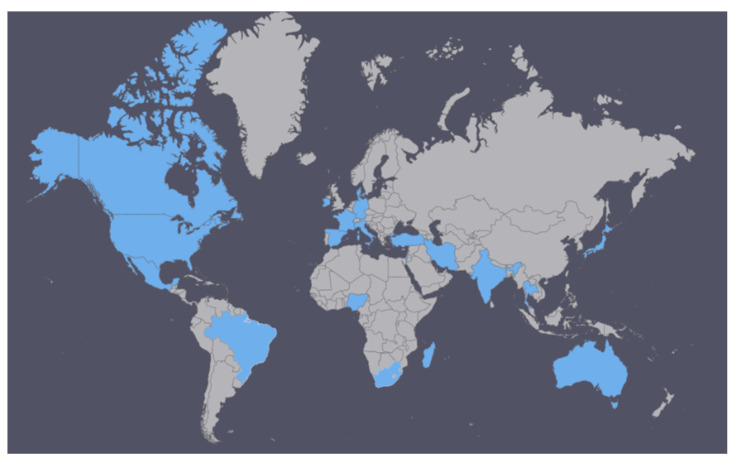
Countries represented in this meta-analysis (in blue).

**Figure A1:**
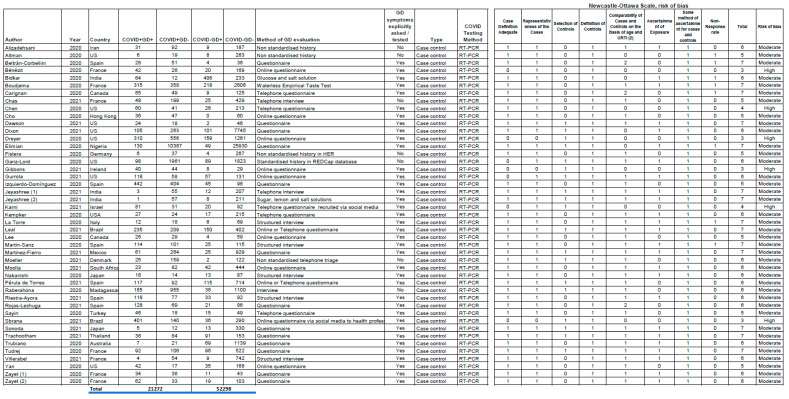
Description of Included Studies and Newcastle-Ottawa Scale Risk of Bias Assessment.

A total of 21,272 COVID-19 positive patients and 52,298 COVID-19 negative patients were included across the 44 studies as seen in Figure A1 and Table A2. The patients were from 21 countries across the major continents, as illustrated in Figure 2.

With reference to Figure A1, all studies utilised RT-PCR as the COVID-19 diagnostic testing method. Most studies collected data regarding GD via questionnaires or structured interviews, except for 3 studies which utilised gustatory testing [139,140,141]. Among the 44 included studies, 7 studies [142,143,144,145,146,147,148] did not test for GD or state that GD symptoms were explicitly asked for.

### 3.2. Risk of Bias

Using the Newcastle-Ottawa scale [51] to assess the risk of bias in each of the included studies, most of the studies were of moderate risk of bias except for 6 studies [34,149,150,151,152,153] which had high risk of bias, as shown in Figure A1. Most studies utilised hospital instead of community controls, failed to control for age as a variable, failed to blind patients and interviewers to the COVID-19 test result during assessment of GD, and failed to report the non-response rate of their study.

### 3.3. Clinical Utility of GD

**Figure 3:**
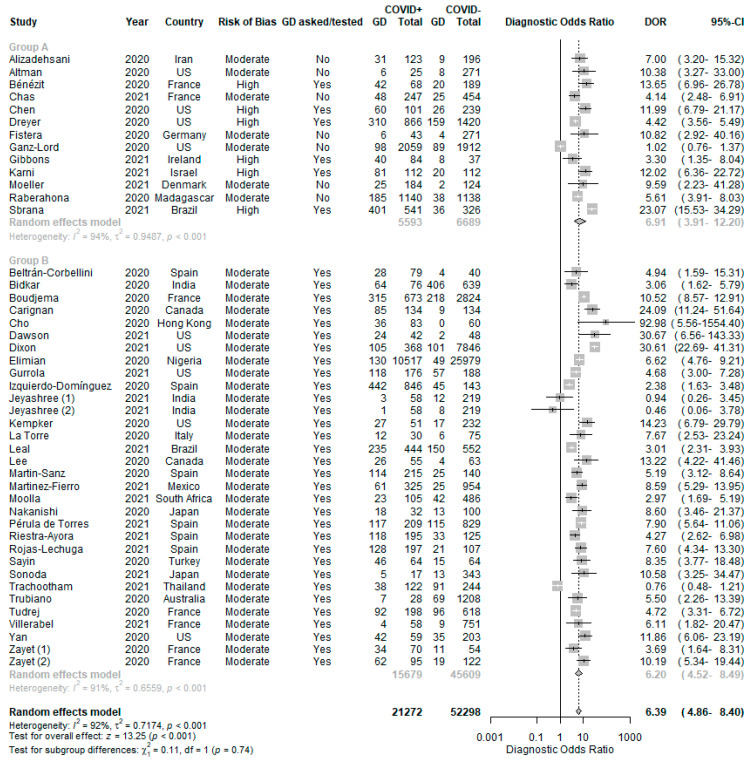
Diagnostic odds ratio of GD in predicting COVID-19 RT-PCR positivity, with Comparison 1—subgroup analysis by risk of bias and if GD was explicitly asked/tested.

**Figure 4:**
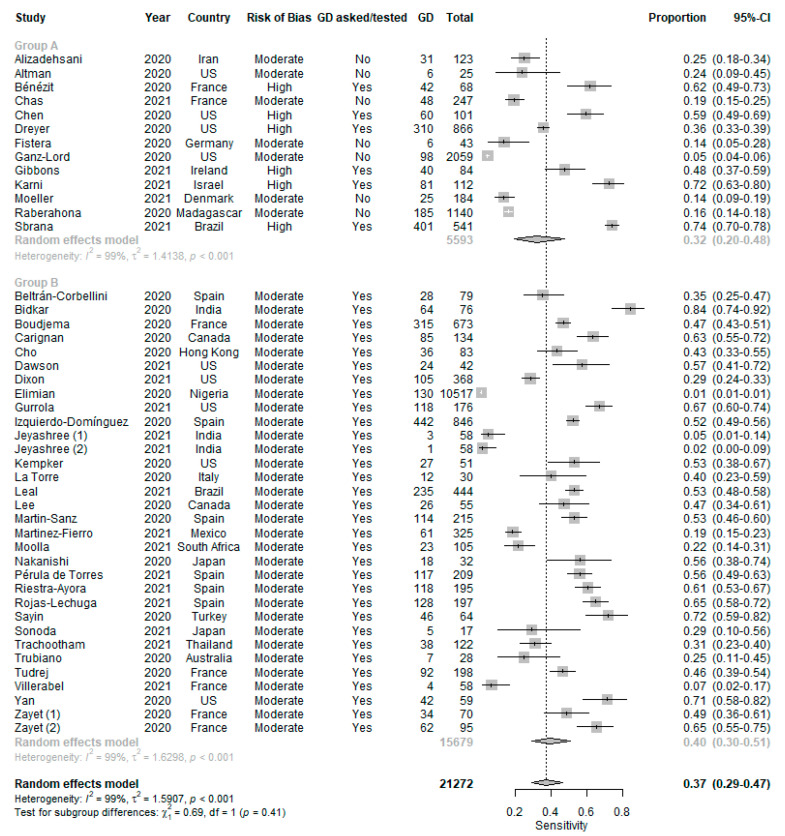
Sensitivity of GD in predicting COVID-19 RT-PCR positivity.

**Figure 5:**
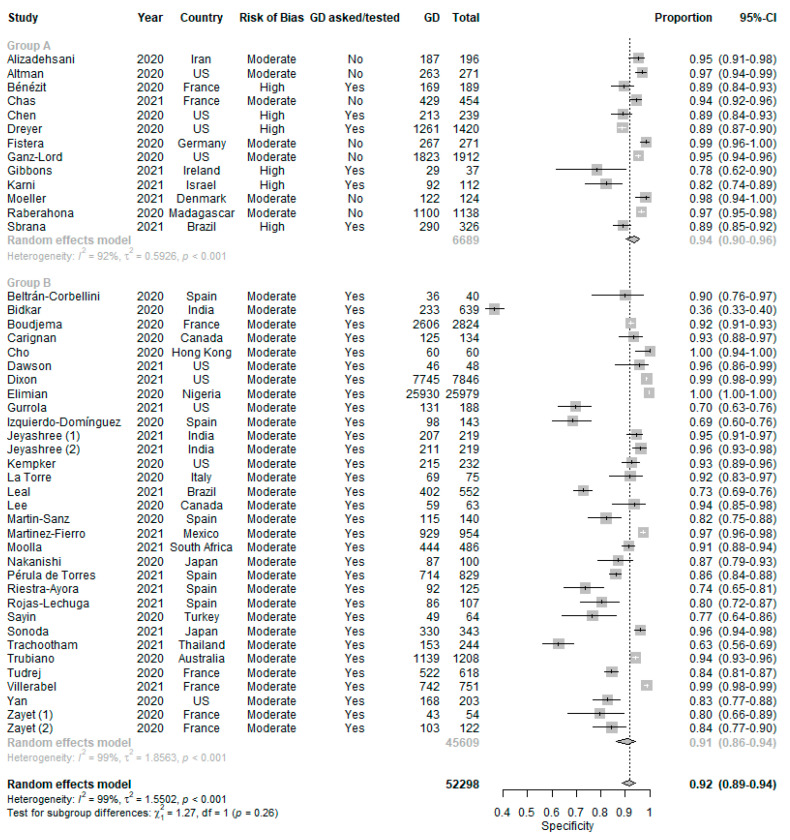
Specificity of GD in predicting COVID-19 RT-PCR positivity.

With reference to Figure 3, patients with GD were more likely to test positive for COVID-19 (DOR 6.39 (4.86–8.40), positive LR 3.84 (3.04–4.84) and negative LR 0.67 (0.64–0.70)). The pooled sensitivity was 0.37 (0.29-0.47) (Figure 4) and the pooled specificity was 0.92 (0.89–0.94) (Figure 5) in using GD to predict COVID-19 RT-PCR positivity. There was significant heterogeneity among the 44 studies (I^2^ = 92%, *p* < 0.01).

Subgroup analysis Comparison 1 failed to show a statistically significant difference between the DOR in Group A as compared to Group B (test for subgroup differences, *p* = 0.74, Figure 3). Among the 31 studies in Group B with low to moderate risk of bias and in which GD symptoms were explicitly asked for or tested, there was still significant heterogeneity (I^2^ = 91%, *p* < 0.01).

**Figure 6:**
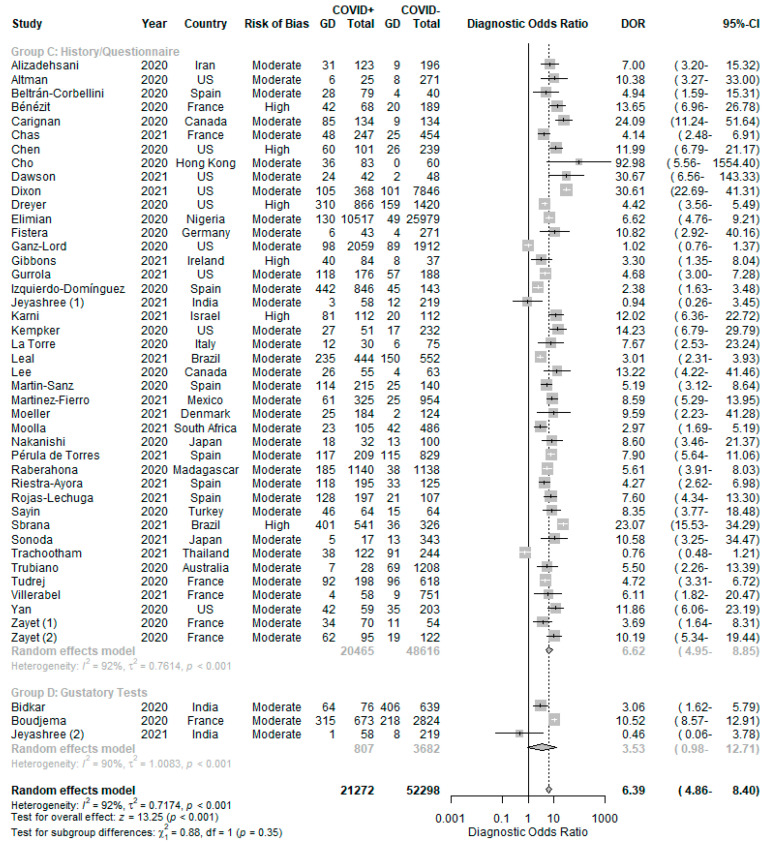
Comparison 2—subgroup analysis by GD assessment method.

Subgroup analysis Comparison 2 (Figure 6) showed that while history/questionnaire-based assessments were predictive of RT-PCR positivity (DOR 6.62 (4.95–8.85)), gustatory testing was not (DOR 3.53 (0.98–12.7)). However, the test for subgroup differences was not statistically significant, *p =* 0.35.

**Figure 7:**
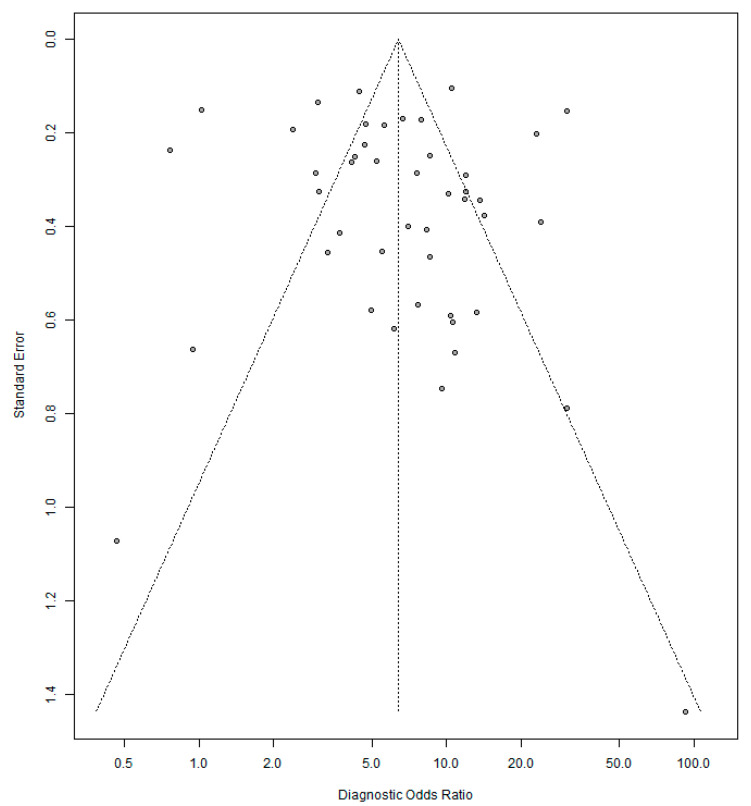
Funnel plot for diagnostic odds ratio of GD in predicting COVID-19 RT-PCR positivity.

The funnel plot shown in Figure 7 and Peters’ test (*p* = 0.61) did not detect the presence of publication bias.

## 4. Discussion

This meta-analysis is the largest study describing the utility of GD in the diagnosis of COVID-19, with 44 included studies, comprising 21,272 COVID-19 positive patients and 52,298 COVID-19 negative controls. It demonstrates that GD as a symptom has high DOR, low sensitivity, high specificity, moderate positive LR and low negative LR in predicting COVID-19 RT-PCR positivity. The DOR of GD was 6.39 (4.86–8.40), lower than that published by Hoang et al. [47] (2 studies, *n* = 519, DOR 12.7 (7.90–20.4)), but similar to that reported in the Cochrane review by Struyf et al. (6 studies, *n* = 9286, DOR 6.60 (5.30 to 8.27)) [154]. Translating this into clinical practice, a patient presenting with upper respiratory tract symptoms and GD likely has COVID-19 and should be quarantined even if the first RT-PCR is negative. However, the absence of GD is insufficient to rule out a COVID-19 infection.

Comparing the clinical utility of GD (with or without OD) to OD (with or without GD) by Pang et al. [49], either GD, OD, or both by Kim et al. [155] in predicting COVID-19 RT-PCR positivity, it can be seen that GD has the lowest DOR and sensitivity, while equivalent specificity. The data in Table 1 suggests that the combination of either GD, OD, or both, may be the best screening criteria, among the 3, to predict COVID-19 RT-PCR positivity.

While GD is useful in predicting COVID-19 RT-PCR positivity, the mechanism by which COVID-19 induces GD is still uncertain. Human angiotensin-converting enzyme 2 (ACE-2) is the entry receptor of SARS-CoV-2 into human cells [156,157]. Using RNA sequencing, ACE-2 has been found to be expressed in the oral cavity, especially in the epithelial cells of the oral tongue [158]. However, a mouse gene expression model thought to be representative of humans, found that ACE-2 is not enriched in most tongue taste bud cells, which suggests that inflammation causing disruption of taste homeostasis, rather than direct viral mediated effects on taste bud cells, is responsible for the GD reported in the literature among COVID-19 patients [159]. One theory is that Toll-like receptors (TLRs) and interferons (IFN) may disrupt normal taste transduction or cell renewal in taste buds [160]. Another theory is that salivary gland dysfunction leads to hyposalivation with subsequent taste impairment [161]. There is also a growing body of evidence that COVID-19 has neuro-invasive potential with positive RT-PCR from cerebrospinal fluid samples [162]. An alternative mechanism of GD is postulated to be cranial nerve VII, IX and X dysfunction with disruption of the central nervous system pathways but this remains controversial [163].

In addition, the optimal method of ascertaining GD remains controversial. Singer-Cornelius et al. [164] suggested that there are large discrepancies between questionnaire-based assessments and gustatory testing, with only 25.6% (10/39) of patients who reported GD demonstrating a measurable deficit on taste strip testing (Burghart Messtechnik GmbH, Wedel, Germany). One possible explanation is the presence of the “ceiling effect” and inability to discriminate subtle levels of GD with taste strips of just four different concentrations [165]. While this problem might be alleviated by the use of extended taste strips testing with additional concentrations [165], it might be time consuming and further increase the risk of exposure to infectious oral secretions. Our study suggests that history/questionnaire-based assessments were predictive of RT-PCR positivity but gustatory testing was not, therefore we propose the former be utilised in assessing GD for the purposes of COVID-19 risk assessment.

Amongst the various screening tools, the use of questionnaires to triage patients into low and high-risk groups for COVID-19 has proven to be effective through different stages of a pandemic. During the initial period of disease outbreak when numbers are high and detection is key, the utility of questionnaires rests in its potential for wide coverage at low costs [111,166]. In January 2020, the first online questionnaire about COVID-19 was launched in China based on early data collected from the initial cases, to stratify the population based on their risk of having COVID-19 and determine the need for further testing or a medical consult. In a span of three weeks, the questionnaire was adopted by all the Chinese provinces and 38 other overseas countries, amassing close to 20,000 responses [166]. Correlating the number of confirmed cases out of these responses facilitated the identification of risk factors for COVID-19 and more importantly, demonstrated how questionnaires could be deployed as a rapid, nationwide screening tool and provide the necessary prompts to particularly high-risk groups for early detection.

Beyond the emergent phase and with international commute resuming amidst COVID-19, questionnaires were adapted as part of travel screening for passengers to fine tune the global response to the pandemic [167]. In the surveillance phase, questionnaires were also used abroad, such as in the US and UK, to gather public perception about the rapidly moving infection and subsequently correct misconceptions through more targeted official press releases [168]. Thus, it is evident that questionnaires have multi-pronged utility. With GD being reported as both a common and possibly early symptom of COVID-19 [169], the inclusion of GD in symptoms-based questionnaires could not only become more relevant as screening tool to aid early detection but also help to educate the public, and allay the distress and functional impact that comes with GD [170].

In the current season where the disease is increasingly being regarded as endemic [171], the move away from gold standard tests with RT-PCR towards self-administered antigen rapid test (ART) kits is testimony to how COVID-19 may progressively be treated akin to a cold. The need for formal testing might be obviated and replaced with either self- or clinician-based clinical diagnosis for isolation and home recovery. For example, Singapore has pioneered a home recovery program (HRP) as the default care arrangement for all COVID-19 patients, unless they belong to a vulnerable age group (80 years and above) or have not completed their vaccinations [172]. HRP now constitutes 40% of daily cases in a bid to reduce the strain on public healthcare inpatient resources [173]. Recovery has become patient-directed with instructions to monitor and upload their vital signs online, while an HRP buddy periodically checks in on their symptoms and progress via telephone calls [174]. The use of self-administered symptom-based questionnaires, featuring GD, may be developed to complement such a recovery program independent of testing. This allows patients to systematically track their clinical progress while offering a potential database of valuable information regarding the clinical course of COVID-19 across demographics and profiles.

A major contributory factor that has permitted countries like Singapore to adopt such methods is their high national vaccination rates and low mortality for COVID-19 patients (estimated to be 0.1% especially for the young and healthy population). However, it has been reported that GD is a possible side effect of COVID-19 vaccinations [175]. In Europe, a small handful of COVID-19 naïve patients reported having new-onset olfactory or taste dysfunction following their COVID-19 vaccinations, but their symptoms lasted for less than two weeks. It is conjectured that post-vaccine inflammation in the olfactory neuroepithelium could contribute to transient olfactory disorder, but there is little established evidence in the current literature [175]. Should GD become a more common or established side effect of vaccinations, whether temporary or permanent, it might confound the use of GD as a potential early screening symptom for COVID-19.

We recognize that there was considerable heterogeneity among the 44 studies in this meta-analysis. Possible sources include: the different populations sampled across 21 countries, lack of a standardised questionnaire in various languages to elicit GD, studies being conducted at different time points of the pandemic (where later studies might be influenced by media coverage of chemosensory dysfunction and COVID-19), some studies assessed GD after COVID-19 testing results were known (recall bias) while others failed to enquire regarding GD symptoms explicitly. The COVID-19 variants, especially the prevalent Delta variant, differ in their virulence, but more importantly, may be associated with less olfactory and gustatory dysfunction. Subgroup analyses attempted to explore some of the above sources of heterogeneity but were not statistically significant.

The limitations of this meta-analysis were an inability to analyse the duration, severity and recovery of GD and possible implications on prognosis due to insufficient data. The studies which were included were of moderate to high risk of bias and failed to control for age and other confounders. This meta-analysis only included studies which were published in English and this resulted in a selection bias as data might not be representative of the non-native English-speaking regions of the world. Future research should be directed towards basic science on the pathophysiology of GD in COVID-19, comparing the performance of various COVID-19 clinical prediction scoring systems and evaluating GD among patients with the different COVID-19 variants.

## 5. Conclusions

GD has high DOR, low sensitivity, high specificity, moderate positive LR and low negative LR in predicting COVID-19 RT-PCR positivity. While the included studies were heterogenous, this meta-analysis provides evidence on the clinical utility of using GD in a screening questionnaire to determine if a patient should undergo further testing, especially in resource-poor regions where COVID-19 testing is scarce. It may also be used to determine the level of clinical suspicion of COVID-19, so that the patient may be advised to quarantine while repeated testing is performed if the initial RT-PCR is negative [30]. There is insufficient evidence to recommend using gustatory testing over questionnaire-based assessment of GD.

## Figures and Tables

**Table 1:** Comparing the clinical utility of GD (with or without OD), OD (with or without GD), either GD, OD, or both, in predicting COVID-19 RT-PCR positivity.

	DOR	Sensitivity	Specificity	Positive LR	Negative LR
GD (with or without OD)	6.39 (4.86–8.40)	0.37 (0.29–0.47)	0.92 (0.89–0.94)	3.84 (3.04–4.84)	0.67 (0.64–0.70)
OD (with or without GD) [49]	11.5 (8.01–16.5)	0.48 (0.40–0.56)	0.93 (0.90–0.96)	6.05 (4.52–8.11)	0.60 (0.54–0.67)
GD and/or OD [155]	10.20 (8.43–12.34)	0.57 (0.47–0.66)	0.91 (0.83–0.96)	Not reported	Not reported

## Data Availability

The authors will share data and the full statistical code upon reasonable request.

## Appendix A

**Table A1:** Search Strategy.

PubMed	(gustat* OR tast* OR dysgeus* OR ageusi* OR parageu* OR “Taste”[Mesh] OR “Taste Perception”[Mesh] OR “Taste Threshold”[Mesh] OR “Taste Disorders”[Mesh] OR “Taste Buds”[Mesh] OR “Dysgeusia”[Mesh] OR “Ageusia”[Mesh]) AND (COVID* OR SARS-CoV-2 OR 2019-nCoV OR coronavirus OR “COVID-19”[Mesh] OR “SARS-CoV-2”[Mesh])
Embase	(gustat* OR tast* OR dysgeus* OR ageusi* OR parageu*) AND (COVID* OR SARS-CoV-2 OR 2019-nCoV OR coronavirus)

## Appendix C

**Table A2:** Citations for Included Studies.

Alizadehsani [142]
Altman [143]
Beltrán-Corbellini [176]
Bénézit [34]
Bidkar [139]
Boudjema [140]
Carignan [177]
Chas [144]
Chen [149]
Cho [178]
Dawson [179]
Dixon [180]
Dreyer [150]
Elimian [181]
Fistera [145]
Ganz-Lord [146]
Gibbons [151]
Gurrola [182]
Izquierdo-Domínguez [183]
Jeyashree (1) [141]
Jeyashree (2) [141]
Karni [152]
Kempker [184]
La Torre [185]
Leal [186]
Lee [187]
Martin-Sanz [188]
Martinez-Fierro [189]
Moeller [147]
Moolla [190]
Nakanishi [191]
Pérula de Torres [192]
Raberahona [148]
Riestra-Ayora [193]
Rojas-Lechuga [194]
Sayin [195]
Sbrana [153]
Sonoda [196]
Trachootham [197]
Trubiano [198]
Tudrej [199]
Villerabel [200]
Yan [201]
Zayet (1) [202]
Zayet (2) [203]

## Appendix D

**Table A3:** List of Abbreviations.

Gustatory dysfunction	GD
Olfactory dysfunction	OD
Reverse transcription polymerase chain reaction	RT-PCR
Odds ratio	OR
Positive likelihood ratio	positive LR
Negative likelihood ratio	negative LR
Preferred Reporting Items for Systematic reviews and Meta-analyses	PRISMA
Upper respiratory tract infection	URTI
COVID+	COVID-19 positive patients
COVID−	COVID-19 negative patients
COVID+GD+	COVID-19 positive patients with gustatory dysfunction
COVID+GD−	COVID-19 positive patients without gustatory dysfunction
COVID−GD+	COVID-19 negative patients with gustatory dysfunction
COVID−GD−	COVID-19 negative patients without gustatory dysfunction

## Appendix E

**Table A4:** PRISMA Checklist [50].

Section and Topic	Item #	Checklist Item	Location Where Item Is Reported
TITLE			
Title	1	Identify the report as a systematic review.	1
ABSTRACT			
Abstract	2	See the PRISMA 2020 for Abstracts checklist.	1
INTRODUCTION			
Rationale	3	Describe the rationale for the review in the context of existing knowledge.	1–2
Objectives	4	Provide an explicit statement of the objective(s) or question(s) the review addresses.	2
METHODS			
Eligibility criteria	5	Specify the inclusion and exclusion criteria for the review and how studies were grouped for the syntheses.	3
Information sources	6	Specify all databases, registers, websites, organisations, reference lists and other sources searched or consulted to identify studies. Specify the date when each source was last searched or consulted.	3
Search strategy	7	Present the full search strategies for all databases, registers and websites, including any filters and limits used.	3, 15
Selection process	8	Specify the methods used to decide whether a study met the inclusion criteria of the review, including how many reviewers screened each record and each report retrieved, whether they worked independently, and if applicable, details of automation tools used in the process.	3
Data collection process	9	Specify the methods used to collect data from reports, including how many reviewers collected data from each report, whether they worked independently, any processes for obtaining or confirming data from study investigators, and if applicable, details of automation tools used in the process.	3
Data items	10a	List and define all outcomes for which data were sought. Specify whether all results that were compatible with each outcome domain in each study were sought (e.g., for all measures, time points, analyses), and if not, the methods used to decide which results to collect.	3
	10b	List and define all other variables for which data were sought (e.g., participant and intervention characteristics, funding sources). Describe any assumptions made about any missing or unclear information.	3
Study risk of bias assessment	11	Specify the methods used to assess risk of bias in the included studies, including details of the tool(s) used, how many reviewers assessed each study and whether they worked independently, and if applicable, details of automation tools used in the process.	3
Effect measures	12	Specify for each outcome the effect measure(s) (e.g., risk ratio, mean difference) used in the synthesis or presentation of results.	3
Synthesis methods	13a	Describe the processes used to decide which studies were eligible for each synthesis (e.g., tabulating the study intervention characteristics and comparing against the planned groups for each synthesis (item #5)).	NA
	13b	Describe any methods required to prepare the data for presentation or synthesis, such as handling of missing summary statistics, or data conversions.	NA (only studies with complete data were included)
	13c	Describe any methods used to tabulate or visually display results of individual studies and syntheses.	3
	13d	Describe any methods used to synthesize results and provide a rationale for the choice(s). If meta-analysis was performed, describe the model(s), method(s) to identify the presence and extent of statistical heterogeneity, and software package(s) used.	3
	13e	Describe any methods used to explore possible causes of heterogeneity among study results (e.g., subgroup analysis, meta-regression).	4
	13f	Describe any sensitivity analyses conducted to assess robustness of the synthesized results.	NA
Reporting bias assessment	14	Describe any methods used to assess risk of bias due to missing results in a synthesis (arising from reporting biases).	3
Certainty assessment	15	Describe any methods used to assess certainty (or confidence) in the body of evidence for an outcome.	NA
RESULTS			
Study selection	16a	Describe the results of the search and selection process, from the number of records identified in the search to the number of studies included in the review, ideally using a flow diagram.	5
	16b	Cite studies that might appear to meet the inclusion criteria, but which were excluded, and explain why they were excluded.	5
Study characteristics	17	Cite each included study and present its characteristics.	16, 17
Risk of bias in studies	18	Present assessments of risk of bias for each included study.	16
Results of individual studies	19	For all outcomes, present, for each study: (a) summary statistics for each group (where appropriate) and (b) an effect estimate and its precision (e.g., confidence/credible interval), ideally using structured tables or plots.	7–10
Results of syntheses	20a	For each synthesis, briefly summarise the characteristics and risk of bias among contributing studies.	6
	20b	Present results of all statistical syntheses conducted. If meta-analysis was done, present for each the summary estimate and its precision (e.g., confidence/credible interval) and measures of statistical heterogeneity. If comparing groups, describe the direction of the effect.	6–10
	20c	Present results of all investigations of possible causes of heterogeneity among study results.	7, 10
	20d	Present results of all sensitivity analyses conducted to assess the robustness of the synthesized results.	NA
Reporting biases	21	Present assessments of risk of bias due to missing results (arising from reporting biases) for each synthesis assessed.	NA
Certainty of evidence	22	Present assessments of certainty (or confidence) in the body of evidence for each outcome assessed.	NA
DISCUSSION			
Discussion	23a	Provide a general interpretation of the results in the context of other evidence.	11
	23b	Discuss any limitations of the evidence included in the review.	13
	23c	Discuss any limitations of the review processes used.	13
	23d	Discuss implications of the results for practice, policy, and future research.	13
OTHER INFORMATION			
Registration and protocol	24a	Provide registration information for the review, including register name and registration number, or state that the review was not registered.	3
	24b	Indicate where the review protocol can be accessed, or state that a protocol was not prepared.	3
	24c	Describe and explain any amendments to information provided at registration or in the protocol.	NA
Support	25	Describe sources of financial or non-financial support for the review, and the role of the funders or sponsors in the review.	14
Competing interests	26	Declare any competing interests of review authors.	14
Availability of data, code and other materials	27	Report which of the following are publicly available and where they can be found: template data collection forms; data extracted from included studies; data used for all analyses; analytic code; any other materials used in the review.	14
